# General Practitioners’ experiences of bereavement care and their educational support needs: a qualitative study

**DOI:** 10.1186/1472-6920-14-59

**Published:** 2014-03-27

**Authors:** Moira O’Connor, Lauren J Breen

**Affiliations:** 1 School of Psychology and Speech Pathology, Faculty of Health Sciences, Curtin University, Perth, WA Australia; 2 School of Psychology and Social Science, Edith Cowan University, Perth, WA Australia

**Keywords:** Bereavement, Grief, General practitioners, Qualitative, Education, Professional development

## Abstract

**Background:**

General Practitioners (GPs) are well-positioned to provide grief support to patients. Most GPs view the provision of bereavement care as an important aspect of their role and the GP is the health professional that many people turn to when they need support. We aimed to explore GPs’ understandings of bereavement care and their education and professional development needs in relation to bereavement care.

**Methods:**

An in-depth qualitative design was adopted using a social constructionist approach as our aims were exploratory and applied. Nineteen GPs (12 women and 7 men) living in Western Australia were interviewed; 14 were based in metropolitan Perth and 5 in rural areas. GPs were invited, via a letter, to participate in a semi-structured interview. The interviews occurred within each GP’s workplace or, for the rural GPs, via telephone, and all interviews were digitally audio-recorded and transcribed.

**Results:**

Analysis was based upon constant comparison and began as soon as possible after each interview. The data revealed four tensions or opposing views concerning bereavement and bereavement care. These were (1) whether grief is a standardised versus an individual process, (2) the role of the GP in intervening versus promoting resilience, (3) the GP as a broker of services versus a service provider, and (4) the need for formal education and professional development versus ‘on-the-job’ experiential learning.

**Conclusions:**

GPs have a critical role in exploring distress, including grief. However, changes need to be made to ensure GPs have up-to-date knowledge of contemporary theories and approaches. GPs urgently need education both at the undergraduate and postgraduate degree levels, and in continuing professional development. Otherwise GPs will rely on out-dated theories and constructions of grief, which may be detrimental to patient care.

## Background

General Practitioners (GPs) are well-positioned to provide grief support to patients [1]. Most GPs view the provision of bereavement care as an important aspect of their role [2] and the GP is the health professional that many people turn to when they need support [3]. Further, patients may visit their GP more frequently following bereavement due to increased morbidity during this time [4,5]. In this context, the role of the GP is twofold; supporting bereaved patients generally, and referring for extra support from a mental health professional when necessary [1,6,7].

In several countries, such as Norway, the United Kingdom, France, Australia and Canada, GPs play a significant role in referring those who need support from a mental health professional [8-10]. In Australia, referral is facilitated by the Better Access to Mental Health Care initiative, which was introduced nation-wide in November, 2006 and allows the GP to refer patients to an allied mental health professional [11]. GPs must see reasonable indicators of mental disorder before they can refer the patient; thus, this system relies heavily on the GPs’ knowledge and experience of mental health generally and, for the purposes of our research, GPs’ awareness and understanding of grief specifically.

GPs have been reported to have a limited awareness of current grief research and tend not to draw upon empirical research findings to inform their work. A UK study of 50 GPs found that they conceptualised grief as linear, stage/phase-based and time-bound, and they had little awareness of contemporary understandings of grief [12]. Current approaches describe grief as unique and multifaceted, and the dual process model articulates that people who are grieving oscillate between confrontation and avoidance of their loss [13]. This lack of knowledge is understandable given that GPs also tend to have limited education to support the provision of bereavement care. Analyses of American, British, and Australian medical courses demonstrate that most presented some information on grief but the information was inadequate and lacked depth or detail (e.g. [14-18]). One recently-developed program on palliative care education for medical students in Germany showed positive effects of the education on students’ self-ratings of competence in communicating with dying patients and their relatives [19]. However, a significant minority of medical students have personal experiences of bereavement [20] and may draw upon these when treating patients, rather than empirical research.

Access to ongoing professional development is also limited. A survey of GPs in the UK found that only 30% of participants had received education about grief, and the majority of participants felt their education to be insufficient [21]. These findings were mirrored in a survey of 320 general practice registrars in the United Kingdom receiving palliative care education, where ongoing grief education and professional development is expected [22]. The authors reported that 67 (20%) participants reported having received no bereavement care training and another 114 (36%) rated their bereavement care training as poor or very poor. GPs report feeling unprepared for providing support to bereaved family members [23]; they rate their knowledge in death, dying, and loss as low; and they acknowledge a clear need for further education and professional development to enhance their knowledge in these areas [24,25]. As such, we aimed to explore GPs’ understandings of bereavement support and their educational and professional development needs in relation to providing bereavement care.

## Methods

### Design

An in-depth qualitative design was adopted using a social constructionist approach as our aims were exploratory and had an applied focus [26]. Social constructionism acknowledges that knowledge is constructed and an understanding of people’s unique experiences is essential to gain an understanding of the issue. We were interested in how GPs construct and conceptualise grief and their interpretations based on their knowledge and experiences of grief.

### Participants

Nineteen GPs (12 women and 7 men) living in Western Australia were interviewed, which enabled us to reach data saturation. The majority (14) were based in metropolitan Perth (population approximately 1.75 million) and 5 worked in rural areas of Western Australia. The number of years in practice ranged from 2 years to 32 years with the majority having over 15 years’ experience. Contact details of GPs were obtained from the Yellow Pages (a listing of addresses and telephone contacts for services in the area). The suburb listing section was used and GP practices were selected from different suburbs in and around the city so that different types of practices were represented. Different sized practices were also contacted. Alongside this, recruitment occurred via liaising with the Perth Divisions of General Practice. Most GPs agreed to be interviewed and those that did not stated they did not have the time.

### Interviews

A semi-structured interview [27] was conducted with each GP. The interviewer was trained and experienced in sensitive interviewing. An interview guide included topics such as the GPs’ education and training in grief; understandings of grief experiences; where they accessed grief information; what they considered best-practice grief treatment and referral; their approach/style of interventions; whether and how bereavement care could be improved; criteria for referral; and the factors they thought would facilitate and impede the incorporation of contemporary literature into their practice. The GPs were asked to provide examples from their experiences with patients to ensure that the data were representative of their practice [12]. The key overarching questions were followed by prompts such as “Can you tell me more about that?” to obtain further information. The mean length of interview was 30 minutes, ranging from 20 minutes to 45 minutes.

### Procedure

GPs were invited, via a letter, to participate in an interview. The interviews occurred within each GP’s workplace or, for the rural GPs, via telephone, and all interviews were digitally audio-recorded. Interviews were transcribed verbatim and checked for accuracy to ensure authentic records for analysis. Ethics approval was obtained from Edith Cowan University’s Human Research Ethics Committee. The GPs received an information sheet and signed a consent form. They were informed that all identifying information, such as the names and practice of the GPs, would be omitted to protect their identities. The GPs were remunerated for their time.

### Analysis

Analysis began as soon as possible after each interview. Analysis began with the reading and re-reading of each transcript and a summary of each was generated. These summaries were compared and contrasted using a process of constant comparison [28]. Codes were collapsed into categories, which were grouped together to examine and isolate meaningful patterns and processes, confirm associations between categories, and to derive specific themes. For example, ‘moving on’ and ‘getting over it’ were coded and collapsed into a category called ‘standardised grief’. This was contrasted with the category of ‘grief as an individualised process’ and identified as a tension. The data collection and analysis occurred concurrently and was guided by the research aims. The research team met regularly throughout data analysis to assist with the development of emerging ideas and to identify alternative interpretations of the text.

Rigour for the study was ensured through addressing the components of credibility, auditability, and fittingness outlined by Beanland et al. [29]. Credibility was ensured through continual immersion in the data before and during analysis, both individually and as a team. An audit trail was maintained via summaries and memos to show how data abstraction and reduction were conducted. Fittingness was achieved by discussing the findings in the light of other research studies in the area and by using the literature to support or refute the concepts emerging from the data during analysis.

## Results

The data revealed four tensions or opposing views concerning understandings of grief, when to intervene, intervention role, and education needs. These tensions were: (1) grief as a standardised versus an individualised process; (2) intervening versus promoting resilience; (3) the GP as a broker of services versus a service provider; and (4) formal education and professional development versus ‘on-the-job’ experiential learning. Each excerpt below is accompanied by the GP identification number.

### Grief as a standardised versus an individualised process

#### Grief as a standardised process

Grief was constructed as time bound, with the expectation that people will ‘get over it’ and ‘move on’ within a set time frame. The first few weeks following the death were perceived as being the most intense emotionally and the first few months as being a period of time when people may withdraw from others:

*The first couple of weeks you expect most people to be crying, you know, feeling pretty low. I’d also expect most people not wanting to see many people and that might go on for the first couple of months*[5].

*Well my understanding is that, and I guess it’s not very educated understanding, but there’s that acute phase. Often it’s quite bad at 3 months*[9].

The stage based approach, particularly that proposed by Elisabeth Kübler-Ross [30], also emerged strongly:

*I tell them about my understanding of the process of bereavement and of the stages. So - initially feeling a bit numb or shocked then numb*[2].

*Well I guess we don’t normally see all the parts of it. Normally we see them quite early on so, yes, a lot of time we see them in sort of the first stage or two. Actually we must have had a little bit of training because there’s kind of a theory of grief – you know, the denial, the bargaining, depression, all those things*[15].

*The Kübler-Ross stages is a typical framework that is used to describe grief. It is quite applicable to most of the patients you see in general practice you know they go through the denial stage and then they go through anger and then they go through acceptance. And that’s a good framework for me to sometimes think about*[4].

Complementing this notion of sequential stages was the notion that many patients become ‘stuck’ in a particular stage rather than emerge from their grief:

*Where she [referring to patient] is at right now is that she is, I mean I don’t have words for stages or anything, but she is still completely in grief. She is angry as well so she is angry that he has passed away and he has left her. She is completely unable to start even looking at living her life without him. So she is still stuck right there and she doesn’t want to go out of the house or anything apart from read her books and feel sad. So she hasn’t moved to any stage of wanting to do anything else. It’s a shame*[8].

In drawing on these stage-based notions as natural and normal, some GPs described other grief experiences as unnatural and abnormal*:*

*[I emphasise] telling them where they are in the process of grieving and loss and what the natural outcomes are for coming through this and that this is a normal process*[10].

*…and my process is really to talk about the grieving and the healthiness and the usual normal process, so that you actually are able to help people move on, so that they don’t become locked in the acute grieving phase. I have got one person who I am seeing at the moment who is actually quite locked in an acute grief reaction despite the death of her mother being 3 years ago. So that is really what we are trying to avoid*[6].

#### Grief as an individualised process

In contrast to viewing grief as a standardised process, there was an acknowledgement by some GPs that people did not go through stages in a set order, or even go through of all the stages:

*I think Kübler-Ross is very instructive in terms of concept. She describes a pathway and a process but it is not always the way that Kübler-Ross described it…A lot of people do follow many of the paths that Kübler-Ross has developed [but] some of them have jumped them and some of them have skipped them*[12].

*You know we, we all quote the Kubler-Ross stages of grief but maybe there’s, there’s new evidence and new paradigms or new thoughts about grief that we aren’t exposed to*[18].

A minority of the GPs conceptualised grief as a unique experience, which varied greatly between bereaved people:

*Well I think first of all to recognise that grief is an individual thing or that people take a different amount of time to go through the process. It’s not the same for everyone…*[1].

*I don’t make any particular judgment about how long it should take them to get over something…You know if they come in after a year and have a cry because it’s an anniversary I don’t think that that is necessarily an abnormal thing*[7].

This tension between grief as a standardised process and grief as an individual process was also demonstrated *within* GPs, with some GPs articulating grief as both standardised and a unique experience. Two GPs articulated a time bounded understanding but then acknowledged that grief does not necessarily happen in that way:

*So grief, when you think about it takes sort of [pause] the classic things is it takes 2 years of that major grieving……..I don’t want to generalise because obviously it’s not the same for everybody*[9].

*…you’ve also got to talk about the time periods too. I mean…yes, grief can be a long process. But I guess if after a couple of week it’s still having a significant impact on their ability to perform their daily activities and normal function then I would, yes, definitely think they should be having some regular counselling……..I mean each person’s really quite different. I mean some people, each person deals with their grief, in different ways. So I think a lot of is actually sort of just trying to guide them as how best they’re going to deal with their grief*[19].

### Intervening versus promoting resilience

#### The need for help

Circumstances that might render bereaved patients to be in greater need of professional support were identified. These circumstances were in relation to the type of death, relationship between the patient and the deceased, the patient’s previous bereavement experiences, and social support available to the patient.

*Well the hardest ones are probably the young ones dying from car accidents or suicide*[10].

*…think if we were going to grade the feeling of loss the worse thing is when a woman has had no problems during pregnancy and then a SID, sudden death syndrome. A child who dies within the first few months of life that to me is so catastrophic*[12].

*I often ask people what their previous experience of grief was because, for a lot of people, it is the first time to encounter a death of somebody close to them. So from that you determine how they might cope with it*[2].

*I saw a lady who was 70 and grieved about 4 years earlier when her husband died of cancer. She nursed him for 2 or 3 years and it was quite a drawn out process. When he died you had the will [referring to legal will] fighting, the crimination and the backstabbing between the family. So her whole bereavement was complicated. Basically her husband’s family wanted to tell her how to nurse him and what to do with him and when he died they contested the will. So she hasn’t been able to grieve properly….it’s taken away the focus from her marriage and relationship that she had with her husband….So not only is she grieving for the loss of her husband but also for the loss of what she thought she had and wasn’t*[11].

*I guess quite a bit comes to how, how much they’ve got close relationships that they’re able to share in*[15].

*I can think of is a woman whose husband died just about 4 or 5 months ago this year. She is probably in her early 70s and she is devastated. She lived her life with him and didn’t really go out anywhere without him the whole time they were together. So she is really struggling to be honest. She’s got no other social supports apart from her kids that visit her once a week. I’ve tried to discuss with her about slowly, when she is ready, moving out into the community maybe volunteering or meeting other people. But she is just completely un-keen*[8].

In these ‘high-risk’ circumstances, some GPs thought that professional intervention was necessary:

*Individually you never get anywhere. We are not islands and we can’t deal with things on our own*[10].

*I think having the person first tap in with a health professional they maybe a GP or the psychologist even it’s important rather than trying to deal with it on their own*[4].

#### Promoting resilience

Conversely, some GPs felt that people generally are resilient and have the tools to cope with bereavement:

*I’ve not had anybody who has really struggled, well, not to my knowledge. I’ve not been aware. I mean it doesn’t seem to be such a huge issue; people do adapt and get on with it. They come to terms with the loss of whoever*[2].

*It’s a part of life experience that people deal with and become resilient in the process of doing so but by doing it themselves as well…*[5].

As such, these GPs emphasised community and family support rather than professional support:

*And some people have lots of other supports and may not need the doctor as much at all*[14].

*So in the event of loss especially someone in the parent’s generation, the mother or father of the extended it’s been interesting to watch how many people are involved in it. It’s been a big experience and even other members within the communities, not necessarily within the Croatian, Italian or Portuguese community but just in the area I’ll hear about a loss or about a bereavement through 3 or 4 different people perhaps before I even hear it from a family member….because there is actually a very tight knit community connection that goes back a long long time. So people who went to school together, people who were neighbours and maybe still are. People who are in businesses but other people have frequented over the years. So it’s been a really interesting thing to watch how many people are involved in that process. It’s like it has a flow on effect all the way out and then all the way back into the centre of the family where the loss has occurred. It’s almost like insulation. It feels like a great deal of cushioning*[7].

### The GP as a broker of services versus a service provider

#### The ‘broker’

GPs described their role as a broker of services where they would refer bereaved patients to other mental health professionals such as psychologists:

*I do that a lot, refer to counselling in general practice….I send a lot of people for grieving off for counselling in the past and it’s been very helpful for them. I’ve got a number of clinical psychologists that I like and would refer [to]. So I would just pick the one that I think would suit the person….In fact almost anyone who had significant grief I would try and refer them because it is such a painful and significant process to get through without help. Most people on that mental health care plan, the 2710 [code for claiming payment for provision of the item], you can find in there depression so you can always find a reason to get them covered for a private clinical psychologist. So that is what I generally do. My criteria would be that I don’t mind the grief being there but it’s the inability to see any movement in their life or that they’re not functioning. Or they are not able to manage their children or even if their grief is overwhelming them and they just need to talk to someone that is a good reason for counselling as well*[8].

In addition to concerns about patients’ ability to engage in daily functioning, referral to mental health professionals was used when the patient was at risk of suicide and self-harm:

*I’d imagine it would be a bit of a team effort so involving psychologists or a psychiatrist if it manifested in terms of anxiety or depression with suicide intent. I don’t know the exact criteria but I suppose if someone seemed to be having [a] prolonged grief reaction or if they had suicidal intent. If there was a co-morbid condition like a depression or an anxiety [disorder]….we have funding under Medicare for psychology, six sessions of counselling by a psychologist….they’re probably my first port of call*[16].

A few GPs expressed less enthusiasm for referral to mental health professionals, because they thought that grief was better managed within patients’ natural social support networks. However, these GPs would still refer to mental health professionals in some circumstances:

*On the whole, it’s basically about staying with family and keeping yourself occupied for a little while….Well there is counselling that I do offer to people and some people find counselling useful but not often. I don’t think it’s very – well, it’s not really good for our clients really. There is one counselling service for Aboriginal people…which is a broad stream counselling service; originally it was for sexual abuse. But certainly if anyone was having trouble with a grief or bereavement that is out of the ordinary I would certainly suggest them. But I have a list of services [looking through service list on pin up board] and if people want I will give them phone numbers and things*[13].

The GPs described several barriers to taking on the role of broker and referring to mental health professionals including bureaucracy, unawareness of resources, liaison with other health care professionals and perceptions of patient adherence:

*[It is difficult] to comply with all the paperwork to go with mental health plans and then [choosing] which items. I’m so paranoid now and actually in 3 years’ time I am very sure all of us who are doing mental health plans are going to be audited on that as well*[3].

*I guess I’m not really aware of many sort of other resources, other than the resource helplines and private counselling*[25].

*Well as a GP it is incredibly difficult to know where to refer them to on the whole*[6].

*….their letters [from clinical psychologists] are not very informative the feedback I get from them, which is a bit of a pity. Now it may be because they don’t want to reveal too much of what the patient said and I can understand that… I guess you don’t want all the stuff that’s been said to be put down on paper … Clinical psychologists tend to just go through the formality of a response and I don’t feel I learn much from them but I think they do help patients*[2].

#### The ‘provider’

In contrast to being a broker for the provision of mental health support for bereaved patients, other GPs saw the provision of psychological support and counselling for mental health issues and bereavement support to be key components of their role:

*I think some GPs are fantastically good at the whole psychological stuff and that would include grief and loss*[11].

*We actually do care for the patient in a holistic manner and that includes their emotional, and, and social wellbeing, their mental health as well as their physical state. So often grief can present as physical symptomatology and we can encompass all of that together*[14].

In viewing the treatment of grief as within the GP remit, there appeared to be a conflation of grief and depression:

*I mean most depression is treated by GPs –it’s not treated by psychologists, it’s not treated by psychiatrists – it’s treated by GPs*[3].

*[There is] a woman whose husband died just about 4 or 5 months ago this year….she is depressed really. I tried to get her to take antidepressants but she won’t do it*[8].

When providing grief support to patients, the GPs described particular strategies drawing on cognitive behaviour therapy and the therapeutic alliance between patient and GP:

*I suppose I try to get them to think positive thoughts. I guess in a way that’s kind of like using cognitive behaviour therapy and trying to change their cognition so “every time you think like this I want you to try and come back to today and what we know today and not what might happen in the future”. So try to bring them back to the now*[1].

*Well I think they [GPs], one thing is that they stick by them…You know, they’re loyal…to their patients. So even though they may not have the words, they’ve got the relationship with their patients and so they often know where they’ve come from and may have known a partner that passed away*[18].

*So it’s not just somebody who is on the outside because you start off with the “remember what happened?” etcetera and go on from there*[5].

*Well I think because GP’s can spend time with patients and they get to see them on a continuing basis, I think they can provide sort of a counsellor or a support for the patient*[16].

### Formal education and professional development versus ‘on-the-job’ experiential learning

#### Need for education and professional development

Most GPs mentioned a lack of formal education in grief or very limited exposure to information about grief:

*It’s something that was not specifically covered [in training]*[1].

*We had a behavioural science unit and there was probably, there may have been one lecture on grief and that’s probably all*[15].

*I didn’t learn anything about dying or bereavement at university. Oh no! That was off limits!*[12].

*We learned Kübler-Ross are those sort of iconic things that, gee it must be the, is it the seventies or eighties that she wrote about those?*[18].

When GPs indicated that they had received education on grief they were usually referring to mental health education generally:

*Depends how you look at it. GPs by definition are trained a lot in mental health and we do a lot of mental health*[3].

*We did Mental Health Level One through the GP training programme but that’s the extent of it… [grief is] not covered particularly. We talk a bit more about depression and referral to community services*[16].

*There’s huge gaps. We get quite a bit of training in depression and stuff but not understanding the difference between grief reactions and depression…and being able to separate that clinically*[15].

*It was more generalised training – identification of various conditions, psychiatric or mental health issues and the management in a general sense, like cognitive behaviour therapy*[1].

*I’ve done lots of mental health training but not specific bereavement grief…I don’t do things, like your average GP, so the norm information gathering for us is through conferences, which may or may not include components such as grief and loss*[11].

#### On-the-job learning

As a result of limited to no training or professional development on grief, experiential, ‘on the job’ learning was the major influence for a minority of the GPs and some felt this was more beneficial than university education or ongoing professional development. The GPs also felt that societal understandings and theories of grief remained the same:

*Most of the stuff I actually learn by applying the principles in terms of the job and getting the feedback from the patient. So in terms of bereavement most of that you will learn on the job by listening openly*[12].

*I don’t think anything changes with grief, loss and bereavement. I don’t think that there are new [ideas] –I just read generally and it’s just part of, on the job. I suppose as you go on and become more experienced, you gain experience in the area. But I don’t think there’s any new treatments. It’s just part of being human and it’s to do with getting more experience*[13].

For some participants this experience was personal and they felt their own experiences of loss and grief made them more empathetic to their patients’ experiences:

*You have to actually feel it and unless you feel it and cry with the pain you’re not learning. You can’t learn about death and dying and grief unless you actually feel it first and understand how you feel and then actually empathise with other people*[12].

*I think you know sometimes, often it’s actually going through grief yourself that you actually make headway and gain greater wisdom in this area*[18].

## Discussion

The GPs described four tensions concerning their understandings of grief, when to intervene, intervention role, and education needs. These tensions demonstrate a lack of clarity and consistency and a ‘piecemeal’ approach to the provision of bereavement care amongst GPs.

The view of grief as a standardised process contrasts with contemporary models of grief as unique and multi-dimensional, and a process that oscillates between loss and restoration [13,31]. These beliefs indicate a lack of knowledge of current models and theories of grief and do not allow for the range of grief experiences and different types of losses. The findings reflect those of Wiles et al. [12], who reported that GPs focussed on grief as a very concrete experience with distinct phases and stages. An expectation that there is a ‘natural’ time to move on could also place stress on people to conform and result in people feeling isolated and stigmatised in a time of vulnerability [32]. The unique, multifaceted nature of grief needs to be emphasised in GP education at all levels so that this conceptualisation of grief becomes the norm rather than the stage-based, time-bound model.

The second tension related to whether the GP sees him/herself as a provider of support or a broker of support involving referral to mental health professionals. This is an interesting paradox in that bereavement, for most people, is a natural event that they will accommodate with minimal support [33]. However, a significant minority will need extra support [34]. The GP is ideally placed to listen and use empathy as they tend to have an ongoing relationship with the patient established over a number of years. They can also ask questions to see if the patient is coping, prescribe pharmacological treatments if deemed necessary, and refer if appropriate (see Lobb et al. [6] for a checklist for GPs to use). However, education is needed to emphasise that the GP does not have to resolve all the issues and that the use of specialised strategies such as cognitive behavioural therapy and counselling require a high level education and training, and timely and appropriate referral to specialist mental health professionals for these approaches may be appropriate [1,7].

Effective communication is essential for both the ‘provider’ and ‘broker’ roles and, again, training would be useful. Community pharmacists face similar issues concerning how to speak to grieving clients when people bring back medications for disposal or want to talk about the death of their family member who may have been using the same community pharmacy for years [35]. It is also important to note that many people may not ask for help or bring up distress without being prompted. Hence, there is a role for education on asking questions [6,36] and eliciting emotional cues [37].

Findings on referral reflect those of Siegal et al. [38] who found that, for general psychological issues, GPs would refer when they felt they had reached the limit of their skills, did not have time to spend with the patient and when they deemed the patient suitable and ready. One barrier to referral these authors identified was that GPs disliked the lack of feedback from mental health professionals. In the current study barriers to referral, such as bureaucracy, liaison with other health care professionals and the time needed for referral, need to be challenged at the systemic level. However, some barriers, such as knowing who to refer to and where to obtain resources, such as lists of appropriate contacts or web pages, could be remedied and improved by education and continuing professional development, and also by simple solutions such as a calendar with key web addresses and telephone numbers. Lobb et al. [6] published a list of key resources in Australia for GPs, which could be updated regularly.

In terms of assessing grief support needs of patients, it is important that GPs acknowledge the circumstances of the death and previous experience with death, and recognise that these factors may affect grief. However, each GP could have a different idea about what is the ‘worst’ type of loss and these conceptualisations could be affected greatly by personal experiences. Complicated or prolonged grief reactions may be more related to background factors such as relationship with the person who has died or attachment style [39,40]. Without adequate education, GPs may overlook these factors and place undue emphasis on situational factors such as type of death or age of the person who died.

The idea of resilience reflects the public health model of grief, which emphasises that most people do not need any extra support other than family or friends, some people need community supports, and a significant minority need access to a mental health professional [34]. Grief education needs to alert GPs to the range of responses. The promotion of resiliance is an ideal approach for the majority of people but education is needed to highlight that, at times, additional support is needed e.g. information on support groups or helplines and, for a minority of people; referral is appropriate [6].

There is a distinct lack of education at the undergraduate degree level for grief and bereavement, and a gap in ongoing professional development in the area. This was mentioned by participants with years of experience as a GP as well as those with less experience. Analyses of American, British, and Australian medical courses demonstrate that most presented some information on grief but the information was inadequate and lacked depth or detail (e.g. [14-18]). This gap needs to be rectified if GPs are to play an active role in supporting bereaved patients and making appropriate and timely referrals.

Some GPs advocated the need to learn from experience and some felt that personal experience of loss was necessary in responding to and supporting others in a sensitive or empathetic way. This latter point contradicts findings from research with palliative care patients, which indicates the need for professional distance and that GPs’ identification with patients may be problematic and have an emotional impact [36]. Limited education in death, dying and grief not only affects the provision of sensitive, timely and appropriate support for bereaved patients; it also affects GPs. Reliance on personal experience could lead to over identification with patients, stress, and ultimately burnout. Education acts as a protective factor when dealing with death and dying [6]. The use of clinical review and case studies could be useful for GPs who prefer experiential learning while protecting them from secondary trauma and burnout [23].

### Limitations and future research

The use of interviews and their systematic analysis provided a contextual and data-driven account of bereavement practice of GPs in Western Australia; this is important as it is the first such study in Australia. However, the findings might not be transferable to GPs working in other contexts. Additionally, we did not collect information concerning each participant’s background, such as ethnic identity or religious affiliation, and these demographics could potentially impact the data and their interpretations. GPs are generalists and busy people and, as a consequence, some interviews were quite short. A convenience sample was used, which means the results may reflect a partial overview. As such, a survey design, based on the findings from the current study, could capture further information from a larger and more representative sample.

## Conclusions

GPs have a critical role in exploring distress [37], including grief [1,2,6]. However, changes need to be made to ensure GPs have up-to-date knowledge of contemporary theories and approaches. GPs urgently need education both at the undergraduate and postgraduate degree levels and in continuing professional development. Otherwise GPs will rely on out-dated theories and constructions of grief as linear, stage/phase-based, and time-bound; and will continue to have little understanding of contemporary models of grief. Alongside this, without education GPs will not realise the limitations of their expertise and will not be able to refer appropriately. Additionally, we need to enhance communication between GPs and mental health professionals and provide easily accessible and useable resources in order to facilitate referral processes and ensure that patients who need extra clinical intervention are able to access appropriate supports. Finally, we need to clarify the processes involved in referring a patient to a mental health professional and also ensure that reimbursement of GPs’ time is adequate in order to optimise patient care.

## Competing interests

No authors have competing financial or other interests.

## Authors’ contributions

MO’C: Contributed to conception and design, conducted analysis and interpretation of data; and wrote the first draft of the manuscript. LB: Developed the project conception and design, contributed substantially to interpretation of data; and was substantially involved in revising the manuscript critically for important intellectual content. Both authors read and approved the final manuscript.

## Pre-publication history

The pre-publication history for this paper can be accessed here:

http://www.biomedcentral.com/1472-6920/14/59/prepub
